# Neurons Release Injured Mitochondria as “Help-Me” Signaling After Ischemic Stroke

**DOI:** 10.3389/fnagi.2022.785761

**Published:** 2022-03-03

**Authors:** Li Gao, Fan Liu, Pin-Pin Hou, Anatol Manaenko, Zhi-Peng Xiao, Fei Wang, Tian-Le Xu, Qin Hu

**Affiliations:** ^1^Central Laboratory, Ren Ji Hospital, Shanghai Jiao Tong University School of Medicine, Shanghai, China; ^2^Cerebrovascular Disease Center, Ren Ji Hospital, Shanghai Jiao Tong University School of Medicine, Shanghai, China; ^3^Department of Anatomy and Physiology, Collaborative Innovation Center for Brain Science, Shanghai Jiao Tong University School of Medicine, Shanghai, China; ^4^Department of Neurology, The First Affiliated Hospital of Chongqing Medical University, Chongqing, China

**Keywords:** ischemic stroke, metabolic stress, mitochondrial release, neuron-glial crosstalk, mitochondrial biogenesis

## Abstract

Mitochondrial dysfunction has been regarded as one of the major contributors of ischemic neuronal death after stroke. Recently, intercellular mitochondrial transfer between different cell types has been widely studied and suggested as a potential therapeutic approach. However, whether mitochondria are involved in the neuron-glia cross-talk following ischemic stroke and the underlying mechanisms have not been explored yet. In this study, we demonstrated that under physiological condition, neurons release few mitochondria into the extracellular space, and the mitochondrial release increased when subjected to the challenges of acidosis, hydrogen peroxide (H_2_O_2_), *N*-methyl-D-aspartate (NMDA), or glutamate. Acidosis reduced the mitochondrial basal respiration and lowered the membrane potential in primary-cultured mouse cortical neurons. These defective mitochondria were prone to be expelled to the extracellular space by the injured neurons, and were engulfed by adjacent astrocytes, leading to increased astrocytic expressions of mitochondrial Rho GTPase 1 (Miro 1) and mitochondrial transcription factor A (TFAM) at mRNA level. In mice subjected to transient focal cerebral ischemia, the number of defective mitochondria in the cerebrospinal fluid increased. Our results suggested that the neuron-derived mitochondria may serve as a “help-me” signaling and mediate the neuron-astrocyte cross-talk following ischemic stroke. Promoting the intercellular mitochondrial transfer by accelerating the neuronal releasing or astrocytic engulfing might be a potential and attractive therapeutic strategy for the treatment of ischemic stroke in the future.

## Introduction

Stroke is one of the major causes of death and permanent disability worldwide, and ischemic stroke accounts for approximately 80% of stroke incidences (Powers et al., 2019). Recombinant tissue plasminogen activator (rt-PA)-mediated thrombolysis is clinically effective after acute ischemic stroke. However, the narrow therapeutic window and the risk of hemorrhagic transformation limit its clinical application (Gao et al., 2020). Therefore, exploring the molecular mechanisms underlying endogenous neuroprotection and finding a novel therapeutic strategy are of great significance for the treatment of ischemic stroke.

Mitochondrial dysfunction is the most immediate response to glucose and oxygen deprivation after ischemia and is closely associated with the early events following ischemic stroke, including reactive oxygen species (ROS)-mediated oxidative stress, acidosis, and *N*-methyl-D-aspartate (NMDA), and glutamate-induced excitotoxicity (Liu et al., 2018; Chen et al., 2020). Numerous studies have evidenced that maintaining the mitochondrial function is essential for the neuronal activity and survival (Huang and Jiang, 2019; Chen et al., 2020). Therefore, mitochondria might be a promising neuroprotective target for the treatment of ischemic stroke.

Recently, intercellular mitochondrial transfer between different cell types, such as mesenchymal stem cell (MSC) and pulmonary alveoli, astrocyte and neuron, and MSC and cardiomyocyte, has been demonstrated and attracted great interest (Plotnikov et al., 2008; Islam et al., 2012; English et al., 2020). Under mitochondrial stress, damaged mitochondria are transported into migrasomes and released to the periphery to facilitate mitocytosis (Jiao et al., 2021). The released mitochondria can also be taken up and reprogrammed by adjacent cells to activate signals for cell survival, highlighting an innovative pathway mediating the pathogenesis of many diseases. The intercellular mitochondrial transfer has also been documented in the cardiovascular injury model and experimental stroke models (Han et al., 2016; Hayakawa et al., 2016; Huang et al., 2016; Liu et al., 2019; Maeda et al., 2020). In ischemic mice, the astrocytes were able to produce functional mitochondria and transfer them into the neurons to promote cell survival and attenuate neurological deficits (Hayakawa et al., 2016) that indicates the existence of a crosstalk between glial cells and neurons during ischemic stroke. However, whether and how the mitochondria of neurons are involved in the neuron-glia cross-talk during ischemic insult has not been explored yet.

## Materials and Methods

### Cell Cultures

Primary neuronal cell cultures were prepared from 1-day-old C57BL/6 pups as described before (Wang et al., 2015). In brief, the cerebral cortex was dissociated and digested with 0.05% trypsin-ethylene diamine tetraacetic acid (trypsin-EDTA) (#E5134, Sigma-Aldrich, St. Louis, MO, USA) for 15 min at 37°C, followed by trituration with fire-polished glass pipettes, and plated in poly-D-lysine-coated culture dishes or 24-well plates. Neurons were cultured with neurobasal medium (#21103049, Thermo Fisher Scientific, Waltham, MA, USA) containing B27% (#17504044, Thermo Fisher Scientific, Waltham, MA, USA) and maintained at 37°C in a humidified 5% carbon dioxide (CO_2_) atmosphere incubator. Cell culture medium (CM) was changed every 3 days and used 10–14 days after seeding. The collected neuronal CM was treated by spin cell debris down with centrifuging at 2,000 rpm for 10 min or by filtrating through a 0.2 μm sterile filter as described previously (Hayakawa et al., 2016) for further experiments.

Primary astrocyte cultures were prepared from the cortex of 16-day-old embryos of C57BL/6 mice. The dissociated cortical cells were seeded on uncoated T75 culture flasks in Dulbecco’s modified eagle medium (DMEM) (#01-055-1A, Biological Industries, Kibbutz Beit Haemek, Israel) supplemented with 10% (v/v) fetal bovine serum (FBS; #SH30070.03, Hyclone Life Science, Logan, UT, USA). After 14 days, the flasks were shaken to remove microglia and passaged with trypsin to reduce the presence of other types of cells. The astrocytes were then detached by trypsinization and plated in six-well plates at a density of 1 × 10^6^ cells/ml.

The Chinese hamster ovary (CHO) cell line was purchased from the Cell Bank/Stem Cell bank (#ACSP-507, Shanghai Chinese Academy of Sciences, Shanghai, China). The cells were cultured in DMEM/nutrient mixture F-12 (#11320-033, Gibco, NY, USA) supplemented with 1% penicillin/streptomycin (#03-031-1B, Biological Industries, Kibbutz Beit Haemek, Israel) and 10% FBS at 37°C in a humidified 5% CO_2_ incubator as described previously (Wu et al., 2018). Cells were grown to 70–80% confluence in 100 mm diameter dishes, and the medium was exchanged every 2 days. The CM was collected, and the mitochondria-depleted CM (mdCM) was obtained by passing the CM through the 0.2 μm sterile filter.

### Establishment of Cerebral Ischemia/Reperfusion Cell Model

Acidosis, ROS-mediated oxidative stress, and excitotoxicity are major events following ischemic stroke. To mimic the cerebral ischemia/reperfusion (I/R) injury *in vitro*, the cultured neurons were exposed to acidosis, hydrogen peroxide (H_2_O_2_), NMDA, and glutamate, respectively. In brief, the neurons were cultured in six-well plates at a density of 1 × 10^6^ cells per well in the neurobasal medium for 10–14 days and used for the subsequent experiments.

The acidosis was stimulated as we reported before (Wang et al., 2020). First, cells were washed three times with a pH 6.5 solution [150 mM sodium chloride (NaCl), 5 mM potassium chloride (KCl), 1 mM magnesium chloride (MgCl_2_), 2 mM calcium chloride (CaCl_2_), and 10 mM glucose, buffered to the desired pH value with 10 mM 4-[2-hydroxyethyl]-1-piperazineethanesulfonic acid (HEPES)] within 5 min at room temperature (22–25°C) and then incubated at 37°C for 1 h. Afterward, the solution was replaced with the normal pH CM and the culture resumed at 37°C for 24 h.

Oxidative stress was induced by H_2_O_2_ stimulation as described previously (Luo et al., 2021). The cells were treated with H_2_O_2_ (#7722-84-1, Sigma-Aldrich, St. Louis, MO, USA) at a concentration of 20 or 50 μM for 1 h. Thereafter, the CM was replaced with normal neurobasal medium for 24 h as restoration.

The NMDA- and glutamate-mediated neurotoxicity was induced as described earlier (Sabogal-Guáqueta et al., 2019). The neurons were challenged to 50 μM NMDA (#M3262, Sigma-Aldrich, St. Louis, MO, USA) or 100 μM glutamate (#G1626, Sigma-Aldrich, St. Louis, MO, USA) for 30 min, and then the original feeding medium was restored for 24 h.

### Mouse Focal Cerebral Ischemia Model

All the experimental protocols were approved by the Animal Care and Use Committee of the Shanghai Jiao Tong University School of Medicine, Shanghai, China. First, 12–14-week-old male C57BL/6 mice (Jiesijie, Shanghai, China) were anesthetized with 1.5–2% isoflurane, and rectal temperatures and cerebral blood flow (CBF) were monitored. Transient middle cerebral artery occlusion (tMCAO) was induced using intraluminal suture as described before (Zuo et al., 2019). A midline neck incision was made, and the left common carotid artery (CCA), external carotid artery (ECA), and internal carotid artery (ICA) were isolated. The ECA was ligated with 5-0 silk suture, and a 6-0 nylon monofilament coated with silicon resin (6023PK, Doccol Corp., Redlands, CA, USA) was inserted from ECA to occlude the MCA. The reduction of the CBF was confirmed by a laser-Doppler flowmeter (moorVMS-LDF-2, Moor Instruments, Devon, UK). Only animals with CBF lower than 20% of baseline were admitted in this study. One hour after occlusion, the suture was gently removed to allow reperfusion. Mice in the Sham group received the same surgical procedure without filament insertion. Body temperature was regularly monitored and maintained at 37 ± 0.5°C with a heating pad. All animals were allowed *ad libitum* access to water and food after surgery.

### Cerebrospinal Fluid Collection and Mitochondrial Tracking

For the collection of cerebrospinal fluid (CSF) (25–50 μl per mice), mice were anesthetized with 2% isoflurane at 24 h post-reperfusion. CSF samples were collected by puncture of the cisterna magna with an A-100 insulin syringe and then stored at -80°C for the detection of mitochondria. For mitochondrial tracking, the CSF was prepared after centrifugation at 2,000 rpm for 10 min. Then, the MitoTracker Green (15 ng/ml, #M7514, Thermo Fisher Scientific, Waltham, MA, USA) was added to the CSF samples (25 μl CSF per mice was diluted with 75 μl PBS) for 1 h at room temperature and then fluorescence-activated cell sorter analysis was performed.

### Adenosine Triphosphate Measurement

Extracellular adenosine triphosphate (ATP) was determined by CellTiter-Glo luminescence (#G7570, Promega, Madison, WI, USA), which can perform cell lysis and generate a luminescent signal that is proportional to the amount of ATP present. Basically, opaque-walled 96-well plates with CM (50 μl) or mdCM (50 μl) were prepared. Then, 50 μl CellTiter-Glo luminescence test solution was added and incubated for 30 min at room temperature. Luminescent signal was recorded using a luminescence microplate reader (BioTek Instruments, Winooski, VT, USA). Since mitochondria produce and restore most of the cellular ATP, the number of released mitochondria in the CM can be estimated by evaluating the extracellular ATP.

### Flow Cytometry

Flow cytometric analysis was performed by the BD LSR II flow cytometer (BD Biosciences, Franklin Lakes, NJ, USA) after the neurons were treated with pH 6.5 and 7.4 solutions for 1 h and reperfusion for 24 h. The CMs were collected from cortical neurons and labeled with MitoTracker Green (#M7514, Thermo Fisher Scientific, Waltham, MA, USA) for 1 h. After being centrifuged at 2,000 rpm for 10 min, the supernatants were used to sort the labeled mitochondria fraction by FACS AriaII cell sorter.

### Mitochondrial Membrane Potential Measurement

For monitoring the health status of mitochondria, JC-1 dye (#T3168, Thermo Fisher Scientific, Waltham, MA, USA) was used, and mitochondrial membrane potential (Δψm) was assessed. The CM and mdCM of cortical neurons and CSF of mice were collected and incubated with JC-1 (5 or 2 μM) for 30 min at 37°C. JC-1 dye exhibits potential-dependent accumulation in mitochondria, indicated by fluorescence emission shift from green (Ex 485 nm/Em 516 nm) to red (Ex 579 nm/Em 599 nm). Δψm was determined by the red/green fluorescence intensity ratio with Synergy H1 Hybrid Multi-Mode Reader (BioTek Instruments, Winooski, VT, USA).

### Oxygen Consumption Rate Analysis

The neurons were cultured in poly-D-lysine-coated 24-well seahorse plates (#100777-004, Agilent, Palo Alto, CA, USA) and exposed to pH 6.5 or 7.4 solution for 1 h. After 24-h restoration, oxygen consumption rate (OCR) was measured according to the vendor recommendation. In brief, neurons were washed twice with XF calibrant solution (#100840-000, Agilent, Palo Alto, CA, USA) and then placed in fresh assay medium. The basal, maximal, and ATP-coupled respiration was monitored after the addition of oligomycin (1 μM, #75351, Sigma-Aldrich, St. Louis, MO, USA), carbonyl cyanide 4-(trifluoromethoxy)phenylhydrazone (FCCP) (1 μM, #2920, Sigma-Aldrich, St. Louis, MO, USA) + sodium pyruvate (5 mM, #P2256, Sigma-Aldrich, St. Louis, MO, USA), antimycin A (4 μM, #A8674, Sigma-Aldrich, St. Louis, MO, USA), and rotenone (1 μM, #R8875, Sigma-Aldrich, St. Louis, MO, USA) as described previously (Savic Azoulay et al., 2020). The assay was performed using Seahorse Bioscience XF96 extracellular flux analyzer (Agilent, Palo Alto, CA, USA), and the statistics were exported from Wave Desktop 2.4 software (Agilent, Palo Alto, CA, USA).

### Immunofluorescence Staining

To investigate the role of the released mitochondria in neuron-glia cross-talk, we labeled the neurons with MitoTracker Red CMXRos (#M22425, Thermo Fisher Scientific, Waltham, MA, USA) and incubated for 1 h. After being washed three times with CM, the neurons were treated with pH 6.5 or 7.4 solution for 1 h. After 24 h, the CM or mdCM was collected and added to the glial fibrillary acidic protein (GFAP)-labeled astrocytes and incubated for 24 h. Then, the astrocytes were washed three times with PBS. The mdCM from neurons was filtered with a 0.2 μm filter and served as control. The astrocytes were fixed with absolute methanol for 10 min on ice and imaged using a fluorescence microscope (Leica, Wetzlar, Germany).

### Real-Time Quantitative-PCR

Total RNA was extracted using TRIzol Reagent (#15596018, Thermo Fisher Scientific, Waltham, MA, USA), and PrimeScript^§^ RT Reagent Kit (#RR037Q, Takara Bio, Tokyo, Japan) was used to obtain cDNA according to the instructions of the manufacturer. The levels of β-actin mRNA were served as an internal reference. The primers used were as follows: mitochondrial Rho GTPase 1 (Miro1) forward: CAAATGAAAGCGGCTGGATAAC; Miro1 reverse: AGCCTAG ATAGCCCAGATACTC; mitochondrial transcription factor A (TFAM) forward: TCCACAGAACAGCTACCCAA; TFAM reverse: CCACAGGGCTGCAATTTTCC; β-actin forward: CCTGGCACCCAGCACAAT; and β-actin reverse: GGGCCGGACTCGTCATAC. All the experiments were performed for six times, and the gene expression was analyzed with the 2^–ΔΔCt^ method.

### Statistical Analysis

Results were expressed as mean ± SD. The *t*-test (normally distributed data) or the Mann-Whitney *U* test (non-normally distributed data) was used to determine the differences between the two groups. One-way ANOVA followed by the Tukey’s Kramer test was used to analyze multiple comparisons. The *p*-value < 0.05 was considered to be statistically significant.

## Results

### Neurons Release Mitochondria Under the Stimulation of Acidosis, Oxidative Stress, and Excitotoxicity

**FIGURE 1:**
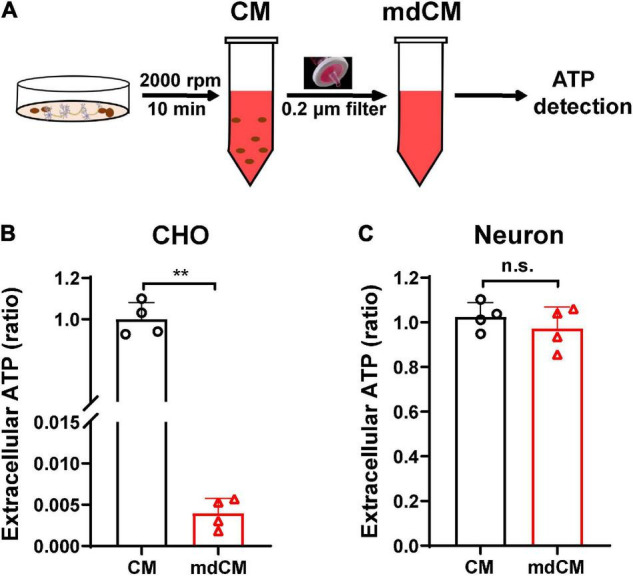
Extracellular ATP detected in the CM and mdCM of CHO cells and neurons. **(A)** The CM was collected by spin cell debris down with centrifuging at 2,000 rpm for 10 min, and the mdCM was collected by passing the CM through the 0.2 μm sterile filter. **(B)** Significant decrease of extracellular ATP concentration in CHO cells was observed after removing mitochondria from the CM of CHO cells. **(C)** No changes in extracellular ATP concentration were observed after removing mitochondria from the CM of neurons. *n* = 4. **p < 0.01 vs. CM. CM, culture medium; mdCM, mitochondria-depleted CM; n.s., not significant.

To assess whether cells release mitochondria into the extracellular space under physiological condition, the CM from CHO cells and neurons were collected. The ATP concentration was measured before or after removing the mitochondria with a 0.2 μm filter (Figure 1A). In CHO cells, the extracellular ATP decreased dramatically after removing mitochondria (Figure 1B, *p* < 0.01 vs. CM). Unlike the CHO cells, there were no significant changes in the extracellular ATP in neurons between before and after being filtered (Figure 1C, *p* = 0.3991).

**FIGURE 2:**
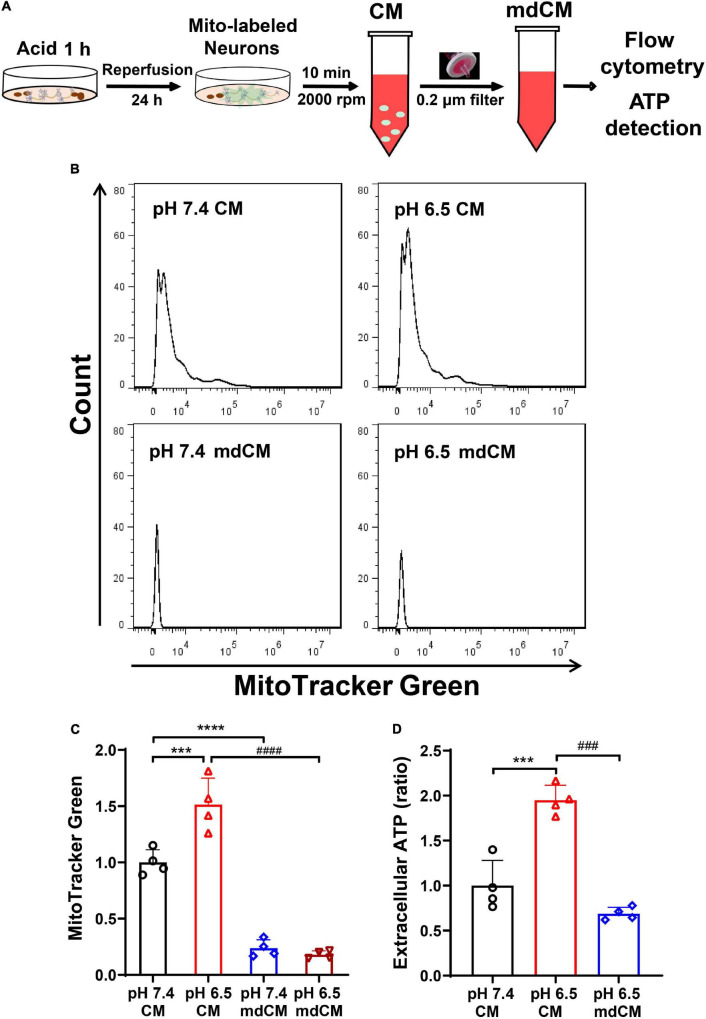
Neurons released mitochondria under the acidosis. **(A)** Schematic diagram describing the experimental procedure. **(B)** The presence of neuronal mitochondria in culture medium (CM) and mitochondria-depleted culture medium (mdCM) in both groups was analyzed by flow cytometry. **(C)** The neuronal mitochondria labeled with MitoTracker Green increased in CM treated with a pH 6.5 solution when compared with the pH 7.4 group, but decreased significantly in mdCM in both pH 7.4 and 6.5 groups. **(D)** The extracellular ATP increased significantly in CM after pH 6.5 treatment, but the ATP concentration decreased dramatically in mdCM after pH 6.5 treatment. *n* = 4. ***p < 0.001, ****p < 0.0001 vs. pH 7.4 CM; ^###^*p* < 0.001, ^####^*p* < 0.0001 vs. pH 6.5 CM.

To investigate whether the neurons release mitochondria under stress, the neurons were labeled with MitoTracker Green and subjected to acidosis with a pH 6.5 solution for 1 h. After restoring for 24 h, the mitochondria in CM and mdCM were examined by flow cytometry (Figure 2A). Treatment with pH 6.5 induced an increase of mitochondria (MitoTracker Green positive) in the CM when compared with the pH 7.4 group. However, the number of mitochondria (MitoTracker Green positive) decreased significantly in mdCM in both groups (Figures 2B,C, *p* < 0.001, *p* < 0.0001 vs. pH 7.4 CM, and *p* < 0.0001 vs. pH 6.5 CM). Consistently, pH 6.5 treatment increased the extracellular ATP in primary-cultured neurons (Figure 2D, *p* < 0.001 vs. pH 7.4 CM), and filtrating through a 0.2 μm filter greatly decreased the ATP level in the pH 6.5 group (Figure 2D *p* < 0.001 vs. pH 6.5 CM).

**FIGURE 3:**
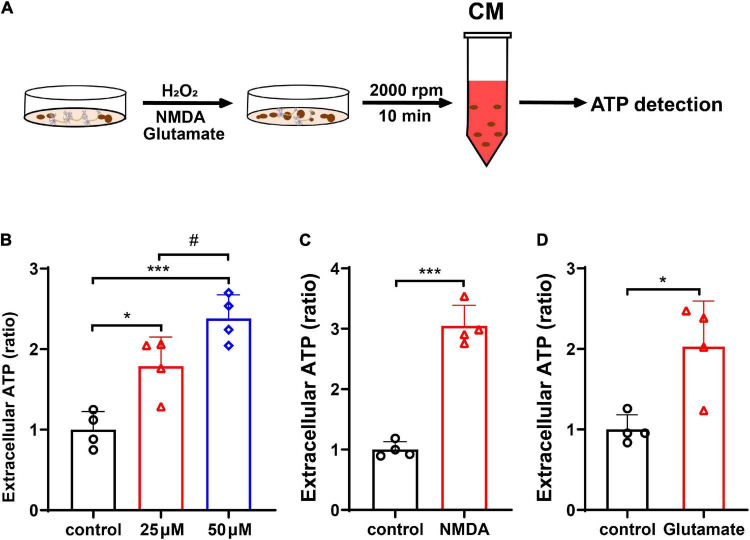
Neurons released mitochondria under hydrogen peroxide (H_2_O_2_)-mediated oxidative stress and glutamate- and *N*-methyl-D-aspartate (NMDA)-induced excitotoxicity. **(A)** The experimental procedure. **(B)** The extracellular ATP increased dose-dependently in CM after H_2_O_2_ treatment. **(C,D)** The extracellular ATP increased significantly after treatment with NMDA (50 μM) or glutamate (100 μM). *n* = 4. *p < 0.05, ***p < 0.001 vs. control, ^#^*p* < 0.05 vs. 25 μM H_2_O_2_.

To verify that neurons release mitochondria under oxidative stress, we assessed the extracellular ATP level in the neuronal CM after H_2_O_2_ treatment. Treatment with H_2_O_2_ at 25 μM dramatically elevated the level of ATP in neuronal CM when compared with control (Figures 3A,B, *p* < 0.05 vs. control); and H_2_O_2_ at 50 μM further increased the release of ATP (Figure 3B, *p* < 0.001 vs. control, *p* < 0.05 vs. 25 μM H_2_O_2_). The effect of glutamate and NMDA excitotoxicity in ATP release was also examined. As shown, exposure of neurons to the NMDA (50 μM) or glutamate (100 μM) resulted in a significant increase of extracellular ATP (Figure 3C, for NMDA, *p* < 0.001 vs. control; and Figure 3D, for glutamate, *p* < 0.05 vs. control).

### Neurons Release Impaired Mitochondria Under Acidosis Stress

**FIGURE 4:**
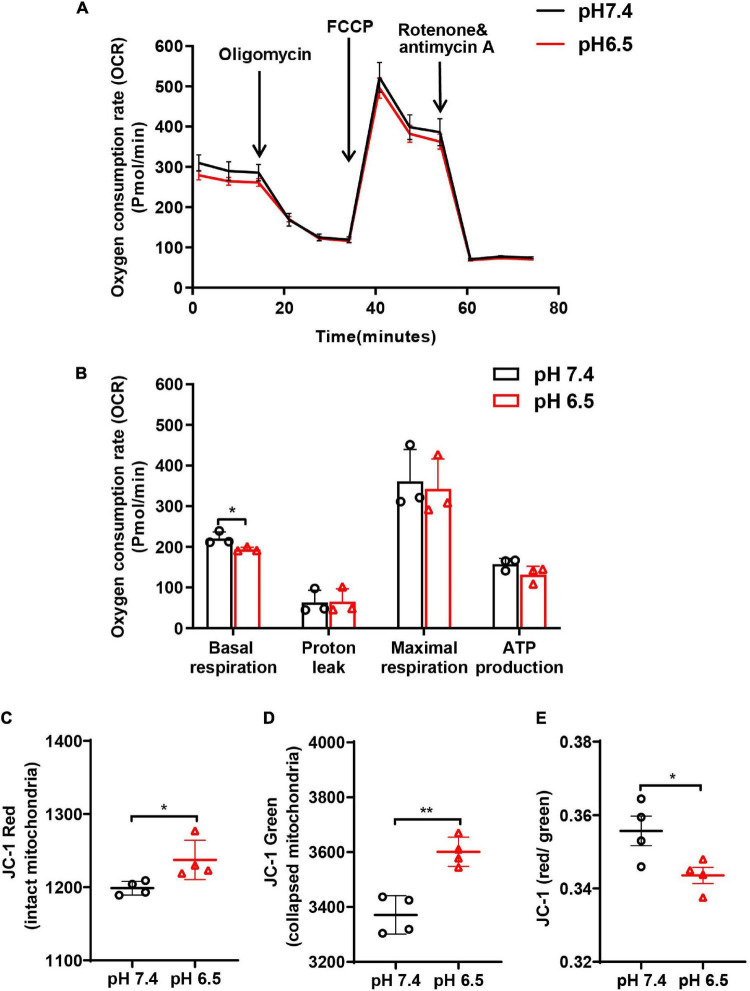
Oxygen consumption rate (OCR) and mitochondrial membrane potential (Δψm; JC-1 red/green ratio) were performed to examine the function of neuronal mitochondria following acidosis. **(A)** Representative OCR traces ± SD measured in neurons treated with pH 6.5 and 7.4 solutions. **(B)** Quantification of basal, “leak,” “maximal” state, and ATP production for pH 6.5- and 7.4-treated neurons. **(C–E)** The JC-1 staining in neurons showed that both JC-1 red and JC-1 green increased after pH 6.5 treatment, but JC-1 red/green ratio decreased significantly. *n* = 4. *p < 0.05, **p < 0.01 vs. pH 7.4.

To analyze whether acidosis compromises mitochondrial respiratory function in neurons, cellular OCR was measured using Seahorse Metabolic Kit. We found that pH 6.5 treatment decreased the basal respiration at 24 h after reperfusion (Figures 4A,B, *p* < 0.05 vs. pH 7.4 group). Then, the mitochondrial respiratory capacity was measured by adding FCCP, which dissipates the Δψm and pushes mitochondria toward their maximal respiration rate. The data showed no difference in the “leak” OCR between the two groups (Figure 4B, *p* = 0.9393). Although the “maximal” OCR respiration and ATP production showed a declining trend after exposing the neurons to pH 6.5 solution, no significant difference was observed when compared to the pH 7.4 group (Figure 4B, for maximal respiration, *p* = 0.7754, and for ATP production, *p* = 0.1458).

To determine the status of the extracellular mitochondria, we monitored Δψm using cationic JC-1 dye. In healthy cells with a normal Δψm, JC-1 is present in the negatively charged interior of mitochondrion to form red fluorescent J-aggregates. In unhealthy or apoptotic cells, due to the decreased Δψm, JC-1 remains monomeric and exhibits green fluorescence. Quantitative analysis showed that after treatment with pH 6.5, both JC-1 red and JC-1 green-labeled mitochondria increased in the CM (Figure 4C, for JC-1 red, *p* < 0.05 vs. pH 7.4; Figure 4D, for JC-1 green, *p* < 0.01 vs. pH 7.4), supporting that acidosis induced mitochondria release. In addition, the ratio of red to green decreased significantly when compared with the pH 7.4 group (Figure 4E, *p* < 0.05).

### Astrocytes Take Up the Neuron-Released Mitochondria

**FIGURE 5:**
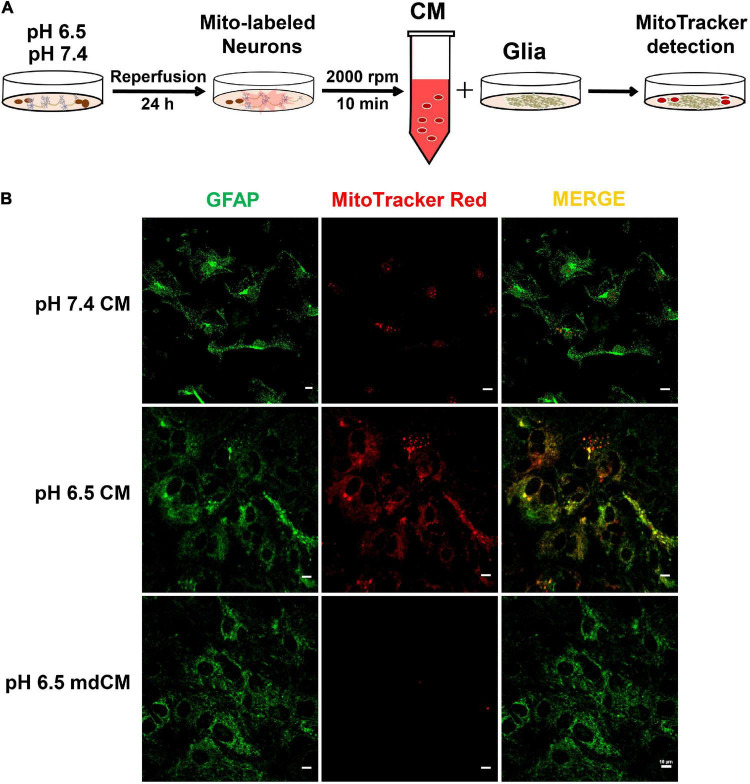
The mitochondria released from neurons were taken up by astrocytes. **(A)** Experimental schematic to detect the cross-talk between neurons and astrocytes after treatment with pH 7.4 and 6.5 solutions. The neurons were labeled by MitoTracker Red CMXRos, and the astrocytes were labeled with GFAP. **(B)** The mitochondria with MitoTracker Red CMXRos were rarely detected within the astrocytes after co-culture with CM from pH 7.4-treated neurons. However, the co-localization of MitoTracker Red CMXRos with GFAP was increased when co-culture with CM from pH 6.5-treated neurons, and the increase was abolished by removing the mitochondria with a 0.2 μm filter.

To determine whether the neuron-released mitochondria are involved in neuron-glia cross-talk after acidosis, the neurons were labeled by MitoTracker Red CMXRos and the astrocytes were labeled with GFAP (green). The neuronal CM from pH 7.4 or 6.5 and mdCM from the pH 6.5 group were collected and cultured with astrocytes for 24 h (Figure 5A). There were few MitoTracker Red CMXRos detected within the astrocytes after co-culture with CM from pH 7.4-treated neurons. However, the presence of MitoTracker Red CMXRos were observed in the astrocytes co-culture with CM from pH 6.5-treated neurons, and removing the mitochondria with a 0.2 μm filter from the neuronal CM reduced the MitoTracker Red CMXRos in the astrocytes (Figure 5B).

### The Enveloped Neuronal Mitochondria Promotes Mitochondrial Biogenesis in Astrocyte

**FIGURE 6:**
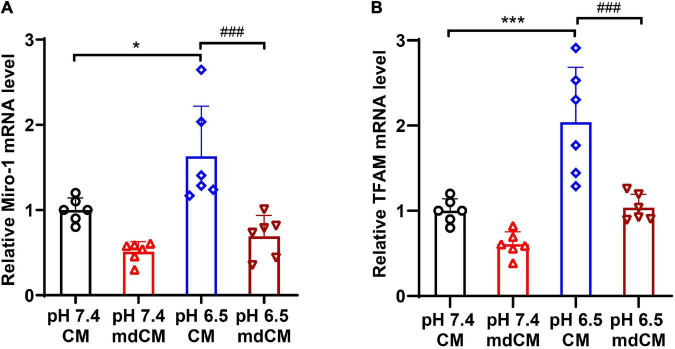
The mRNA expressions of Miro1 and TFAM in astrocytes co-cultured with CM and mdCM from pH 7.4- and 6.5-treated neurons. The mRNA level of Miro1 **(A)** and TFAM **(B)** increased significantly in the CM from pH 6.5-treated group but no significant difference in the mdCM group. *n* = 6. *p < 0.05, ***p < 0.001 vs. pH7.4 CM; ^###^*p* < 0.001 vs. pH 6.5 mdCM. CM, culture medium; mdCM, mitochondria-depleted CM; Miro-1, mitochondrial Rho GTPase 1; TFAM, mitochondrial transcription factor A.

To explore the mitochondrial biogenesis in astrocytes after engulfing the neuronal mitochondria, the mRNA expressions of Miro1, and TFAM in the astrocytes were determined. The level of Miro1 mRNA and TFAM mRNA increased significantly in the astrocytes cultured with CM from pH 6.5-treated neurons when compared to CM from the pH 7.4 group (Figures 6A,B, *p* < 0.05, *p* < 0.001 vs. pH 7.4 CM). However, the mRNA expressions of Miro1 and TFAM decreased significantly in the mitochondria depleted (md) pH 6.5-treated group (Figures 6A,B, *p* < 0.001 vs. pH 6.5 CM).

### The Damaged Mitochondria Increased in Cerebrospinal Fluid After Middle Cerebral Artery Occlusion in Mice

**FIGURE 7:**
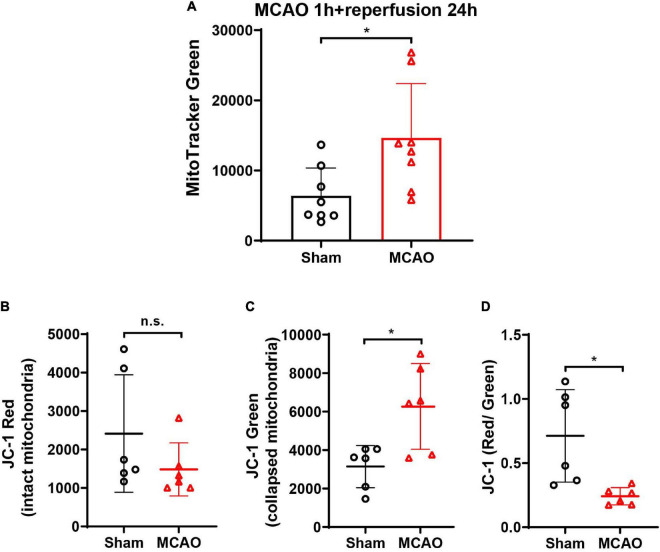
The detection of mitochondria and Δψm (JC-1 red/green ratio) in the CSF of MCAO mice. **(A)** After cerebral ischemia, the MitoTracker Green-labeled mitochondria elevated significantly in CSF at 24-h post-reperfusion. *n* = 8. *p < 0.05 vs. Sham. **(B)** The JC-1 staining in CSF showed no significant difference of JC-1 red between the Sham and MCAO groups. **(C)** JC-1 green increased significantly in the CSF after MCAO when compared with the Sham group. **(D)** JC-1 red/green ratio decreased significantly in the CSF after MCAO. *n* = 6. *p < 0.05 vs. Sham. CSF, cerebrospinal fluid; MCAO, middle cerebral artery occlusion; n.s., not significant.

To investigate whether neurons release mitochondria after ischemic stroke *in vivo*, the MCAO model was established in mice. After being labeled with MitoTracker Green, the content of mitochondria in CSF was detected by flow cytometry. The data showed that when compared with animals in the Sham group, MCAO induced a great increase of mitochondria (MitoTracker Green) in the CSF at 24-h post-reperfusion (Figure 7A, *p* < 0.05 vs. Sham group), suggesting that ischemic stroke-induced massive death of brain cells leading to the release of mitochondria in extracellular space and CSF.

To determine the status of the released mitochondria in CSF, JC-1 dye was used to monitor Δψm. MCAO did not increase the JC-1 red mitochondria in CSF when compared with the Sham group (Figure 7B, *p* = 0.1320 vs. Sham group). However, the JC-1 green mitochondria increased significantly after MCAO (Figure 7C, *p* < 0.05 vs. Sham group), indicating that ischemic stroke increased the mitochondria release. Furthermore, the ratio of red to green decreased significantly when compared with the Sham group (Figure 7D, *p* < 0.05 vs. Sham group), suggesting that the damaged mitochondria were more easily to be released after MCAO.

## Discussion

**FIGURE 8:**
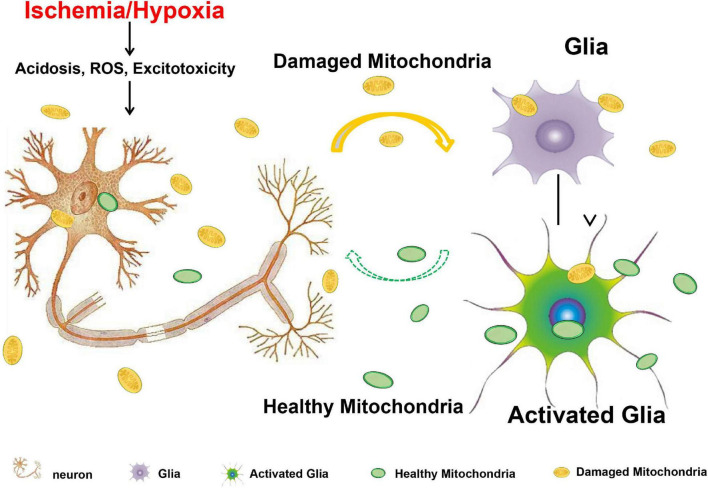
Acidosis, oxidative stress, and excitotoxicity are closely associated with mitochondrial dysfunction and are the major culprits that contribute to the ischemic neuronal death after stroke. When neurons are confronting with these harmful challenges, they might release the defective mitochondria to act as a “help-me” signaling, and recruit the adjacent astrocytes for energy support by promoting astrocytic mitochondrial biogenesis. Solid arrow indicates the confirmed results from this study, and dashed arrow indicates the assumption from previously published studies.

In this study, we investigated the neuronal mitochondria release under hypoxia/ischemia insult and the mitochondria-mediated cross-talk between neurons and astrocytes. We found that compared to CHO cells, neurons under physiological condition seldom release mitochondria. Neurons were, however, prone to release the defective mitochondria when challenged with acidosis, H_2_O_2_-induced oxidative stress, or glutamate- and NMDA-induced excitotoxicity. In MCAO mice, increased mitochondria were observed in the CSF. In addition, we found that the damaged mitochondria can be engulfed by the astrocytes and induced the increase of Miro1 and TFAM at the mRNA level, suggesting the activation of mitochondrial biogenesis in the astrocytes. We concluded that when confronting with metabolic stresses, neurons might release the defective mitochondria to act as a “help-me” signaling and recruit the adjacent astrocytes for energy support by promoting astrocytic mitochondrial biogenesis (Figure 8). Our findings suggested that facilitating the defective mitochondrial releasing and astrocytic mitochondrial engulfing might be a potential therapeutic strategy for the neuroprotection in ischemic stroke.

Due to the high energetic demand, neurons are particularly susceptible to hypoxic and ischemic insult. Following ischemic stroke, the depletion of oxygen and glucose compromises the mitochondrial metabolism and depolarized mitochondrial membranes, leading to excessive calcium accumulation, overproduction of ROS, and activation of programmed cell death (Liu et al., 2018; Chen et al., 2020). Clearance of the dysfunctional mitochondria is critical for maintaining the viability of neurons and, subsequently, for neurological function recovery after ischemic stroke. Recently, accumulating evidence has demonstrated that intercellular mitochondrial transfer occurs in physiological condition and diseases (Davis et al., 2014; Liu et al., 2014; Hayakawa et al., 2016; Jiao et al., 2021). The extracellular mitochondria can be taken up by adjacent cells, which leads to the reprogramming of the cell metabolism and to relaying the signals for cell survival. Therefore, mitochondria itself may act as transducers undertaking metabolic cross-talk between injured cells and healthy cells. In this study, we found that CHO cells released mitochondria into the extracellular space. Compared with CHO cells, neurons under physiological condition release fewer mitochondria, probably because neurons are high energy consuming and depend more on mitochondria for energy production. When the neurons were exposed to acidosis, H_2_O_2_, glutamate, or NMDA excitotoxicity, the neuronal mitochondria decreased the level of basal respiration and lowered the membrane potential. Neurons dispose these defective mitochondria by releasing them into the CM. It is known that healthy mitochondria are crucial for cell viability. In contrast to the events referred to those characterised for damaged mitochondria such as cytochrome *c* release and caspase activators, changes in electron transport, and/or loss of mitochondrial transmembrane potential result in activation of programmed cell death pathways. We suggested, therefore, that under toxic stresses, neurons may be prone to expel the damaged mitochondria to sustain cell viability. This observation is in line with the recent study that under mitochondrial stress, mitochondria with low Δψm were shed off from the cell body by migrasomes (Jiao et al., 2021).

It has been demonstrated that mitochondria are critical players in intercellular communications. Cells secrete mitochondrial proteins and mtDNA into their environment and interact with adjacent cells to participate in metabolic regulation or immune system stimulation (Todkar et al., 2021). Mitochondrial DNA, *N*-formyl peptides, and membrane component cardiolipin are important damage-associated molecular patterns and induce innate immune responses (West et al., 2011; Riley and Tait, 2020). Therefore, discarding the damaged mitochondria extracellularly is not a desirable solution. Interestingly, Davis et al. (2014) reported that retinal neurons could release damaged mitochondria and transfer them to astrocytes for disposal and recycling in the optic nerve head. Mahrouf-Yorgov et al. (2017) demonstrated that mitochondria from injured endothelial cells can be engulfed and degraded by MSCs, which lead to the activation of cytoprotective enzyme, i.e., heme oxygenase-1 and promotion of mitochondrial biogenesis. These two studies suggested that the damaged mitochondria released from dying cells not only serve as a disposal but also as a key rescue signal that recruiting the adjacent cells as well as recycling the mitochondria for quickly counteracting the energy deficits. Mitochondrial transfer has also been demonstrated between neurons and astrocytes to promote neuroprotection and neural recovery following ischemic stroke. Under the ischemic condition, astrocytes transferred functional mitochondria into the neighboring neurons and provided endogenous neuroprotective effects *in vivo* and *in vitro* (Hayakawa et al., 2016). Inhibition of astrocytic mitochondrial function suppressed their ability to protect neurons against excitotoxicity and make neurons vulnerable to ischemia (Voloboueva et al., 2007). Here, we presented that after being subjected to harmful stimulus, neurons released the defective mitochondria into extracellular space. The released mitochondria could be taken up by adjacent astrocytes that would explain the increased expressions of Miro1 and TFAM at mRNA level in astrocytes, which was observed in our publication. Therefore, we hypothesized that the neuron-released mitochondria serve as a “help-me” signaling and mediate the neuron-astrocyte cross-talk following ischemic stroke (Figure 8).

We observed that mitochondria with low membrane potential were upregulated in CSF of the mice subjected to focal cerebral ischemia, which was in accordance with previous publications (Hayakawa et al., 2016). The extracellular mitochondria were also detected in CSF with decreased Δψms in subarachnoid hemorrhage (SAH) rats (Chou et al., 2017). In the clinical studies, extracellular mitochondria were also found in the CSF from patients with SAH, and the higher Δψm was correlated with good clinical recovery (Chou et al., 2017). Extracellular mitochondria in CSF may be a potential biomarker for the diagnosis and prognosis of acute stroke.

## Conclusion

We presented that when subjected to harmful stimulus, neurons release the defective mitochondria into extracellular space. The released mitochondria could be taken up by adjacent astrocytes to activate the mitochondrial biogenesis. Our findings suggested that neurons may release the defective mitochondria as a “help-me” signaling to mediate the neuron-astrocyte cross-talk following ischemic stroke. Boosting the recycling of endogenous mitochondria between neurons and astrocytes might be a potential therapeutic strategy to counteract the energy deficiency in acute ischemic stroke.

## Data Availability Statement

The original contributions presented in the study are included in the article/Supplementary Material, further inquiries can be directed to the corresponding authors.

## Ethics Statement

The animal study was reviewed and approved by the Animal Care and Use Committee of Shanghai Jiao Tong University School of Medicine, Shanghai, China.

## Author Contributions

LG, FL, T-LX, and QH conceived, designed, and coordinated this study. LG, FL, and P-PH performed the experiments and analyzed the data. Z-PX and FW helped with cell culture and performed the RT-qPCR. LG, AM, T-LX, and QH wrote, revised, and checked the data analysis. All the authors revised and approved the final version of the manuscript.

## Conflict of Interest

The authors declare that the research was conducted in the absence of any commercial or financial relationships that could be construed as a potential conflict of interest.

## Publisher’s Note

All claims expressed in this article are solely those of the authors and do not necessarily represent those of their affiliated organizations, or those of the publisher, the editors and the reviewers. Any product that may be evaluated in this article, or claim that may be made by its manufacturer, is not guaranteed or endorsed by the publisher.
